# Robustness analysis for quantitative assessment of vaccination effects and SARS-CoV-2 lineages in Italy

**DOI:** 10.1186/s12879-022-07395-2

**Published:** 2022-04-29

**Authors:** Chiara Antonini, Sara Calandrini, Fortunato Bianconi

**Affiliations:** 1 ICT4Life srl, Perugia, Italy; 2 Department of Engineering, University of Perugia, Perugia, Italy; 3 COVID-19 Epidemiological Unit, Regional Government of Umbria, Perugia, Italy

**Keywords:** COVID-19, Conditional robustness analysis, ODE model, Italy

## Abstract

**Background:**

In Italy, the beginning of 2021 was characterized by the emergence of new variants of SARS-CoV-2 and by the availability of effective vaccines that contributed to the mitigation of non-pharmaceutical interventions and to the avoidance of hospital collapse.

**Methods:**

We analyzed the COVID-19 propagation in Italy starting from September 2021 with a Susceptible-Exposed-Infected-Recovered (SEIR) model that takes into account SARS-CoV-2 lineages, intervention measures and efficacious vaccines. The model was calibrated with the Bayesian method Conditional Robust Calibration (CRC) using COVID-19 data from September 2020 to May 2021. Here, we apply the Conditional Robustness Analysis (CRA) algorithm to the calibrated model in order to identify model parameters that most affect the epidemic diffusion in the long-term scenario. We focus our attention on vaccination and intervention parameters, which are the key parameters for long-term solutions for epidemic control.

**Results:**

Our model successfully describes the presence of new variants and the impact of vaccinations and non-pharmaceutical interventions in the Italian scenario. The CRA analysis reveals that vaccine efficacy and waning immunity play a crucial role for pandemic control, together with asymptomatic transmission. Moreover, even though the presence of variants may impair vaccine effectiveness, virus transmission can be kept low with a constant vaccination rate and low restriction levels.

**Conclusions:**

In the long term, a policy of booster vaccinations together with contact tracing and testing will be key strategies for the containment of SARS-CoV-2 spread.

**Supplementary Information:**

The online version contains supplementary material available at 10.1186/s12879-022-07395-2.

## Background

The COVID-19 pandemic is a global emergency that caused not only an health crisis but also an economic and social one. The severe acute respiratory syndrome coronavirus 2 (SARS-CoV-2), the virus responsible of COVID-19, has spread all over the world, producing millions of hospitalizations and deaths and a significant economic damage.

During the first and second waves of the COVID-19 pandemic, non-pharmaceutical interventions (NPIs) were the only available measures to reduce the burden on healthcare systems and save lives. As of December 2020, multiple vaccines against COVID-19 were approved but travel restrictions and social distancing are still needed, especially because of the spreading of highly transmissible variants of concern (VOC) [1].

In Europe, Italy was the first country severely hit by COVID-19 since March 2020. In Italy, the virus initially spread to the northern regions, forcing the Italian Government to carry out a total lockdown from March to May 2020 [2]. When the lockdown was released, tourist flows, together with school reopening and social events caused a new wave of infections with a peak in November 2020 [3]. For social and economical reasons, the lockdown adopted in the second wave was less restrictive than the first one. In this phase, the Italian Government adopted an area-specific system of policies, by dividing regions in different colors based on the local risk spread [4]. At the beginning of 2021, new COVID-19 variants were detected while the mass vaccination campaign was getting underway [5, 6]. High priority for vaccination was given to hospital workers and nursing home staff. Then, the vaccination plan continued based on the most advanced age up to the youngest, giving priority to fragile people. To date, the Anti COVID-19 Vaccines Report states that 44.200.401 people have completed the vaccination cycle which represent the 81.84% of the population over 12 [7]. As regards the spread of variants, the highly contagious Delta variant, identified in India in December 2020, has gained dominance in Italy and now accounts for about 99.99% of total cases [8]. In order to avoid another COVID-19 infection resurgence, the Italian Government, following the European Union, introduced the COVID-19 vaccination pass (VP). Initially, VPs were required mainly for working at school, traveling on trains and flights and participating at crowded events. From October 15, they are compulsory for all workers in the public and private sector [9].

In this context, epidemiological models based on COVID-19 data can provide useful insights into the impact of the vaccination campaign, of variants spread and the employment of NPIs. Many models have been developed in order to understand COVID-19 dynamics in multiple countries and inform policy decisions [10]. Current studies are mainly based on the well-known Susceptible Infected-Recovered (SIR) model, which describes the human-to-human transmission through Ordinary Differential Equations (ODEs).

Here, our main scope is to study the time-varying COVID-19 propagation in Italy, under the presence of VOCs and of vaccines with different efficacy. To address that, we apply the Conditional Robustness Analysis (CRA) algorithm to a Susceptible-Exposed-Infected-Recovered (SEIR) model which includes intervention measures and the administration of vaccines (SEIRL-V). The CRA is a method for identifying model parameters yielding a desired system behavior and, for this reason, it is particularly suitable for analyzing how each epidemiological and vaccination parameter affects epidemic diffusion. The model has already been employed to represent the COVID-19 epidemic in Italy and in the Umbria region, one of the first Italian regions overwhelmed by the spread of new variants [11, 12]. The model has already been calibrated using the Bayesian method Conditional Robust Calibration (CRC) against COVID-19 data of hospitalized, intensive care unit (ICU) and dead patients [13–15]. Through application of the CRA, we aim at detecting key parameters for the containment of SARS-CoV-2 spread and propose a quantitative framework for planning long-term strategies.

## Methods

**Fig. 1:**
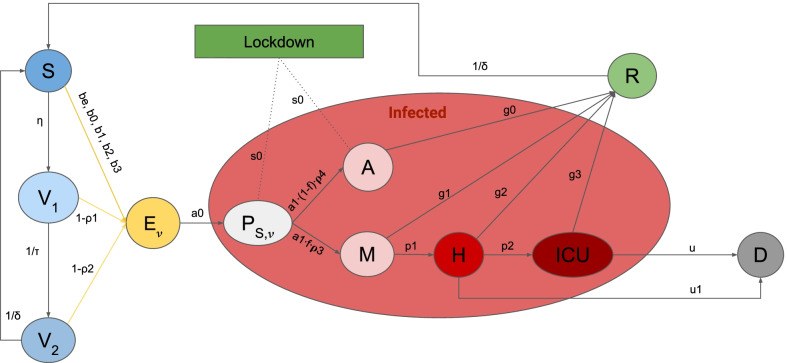
Flowchart of the computational workflow adopted to study the spread of COVID-19. The ODE model is calibrated against epidemiological data of COVID-19 through the Bayesian method CRC. Then, the CRA algorithm is applied in order to study the influence of infection and vaccination parameters on the hospitalization capacity. The main steps of the CRA are the following: (1) choice of an evaluation function representative of the property of interest, (2) perturbation of the parameter space with LHS, (3) model integration using the generated parameter vectors, (4) kernel density approach for estimating the conditional density of each model parameter. The final result is an index, called MIRI, which denotes the impact of the parameter on the chosen model observable

We use an extended SEIR model which includes vaccination and intervention measures, in order to study the evolution of COVID-19 in Italy. A description of the workflow applied to the model is shown in Fig. 1. First of all, the model is calibrated against Italian data of hospitalized, ICU and dead patients, available in the GitHub repository of the Italian Civil Protection Department [16]. Since the number of detected cases can be biased by testing protocols, we rely only the number of hospital and ICU admissions over time and on the number of deaths instead of new positive cases. The repository contains Italian data from the beginning of the pandemic until today but we considered only COVID-19 data from September 2020 to May 2021. During this time span, in Italy, as in the rest of the world, the presence of a new variant, known as the B.1.1.7 lineage, was detected and caused a new peak of infections in late March 2021 [17]. To take into account the higher transmissibility of this variant, we varied the transmission rate parameters of the SEIR model. The method used for parameter estimation is CRC, which is a variant of the class of Approximate Bayesian Computation Sequential Monte Carlo (ABC-SMC) algorithms. Results of the application of CRC to the SEIR model are shown in Additional file 1: Fig. S1, which depicts the behavior of hospitalized, ICU and D patients in comparison with available data. Then, on the calibrated model, we run the CRA algorithm through our CRA Toolbox, in order to quantify the influence of all parameters on the hospitalization capacity and logistics.

### Description of the SEIRL-V model

**Fig. 2:**
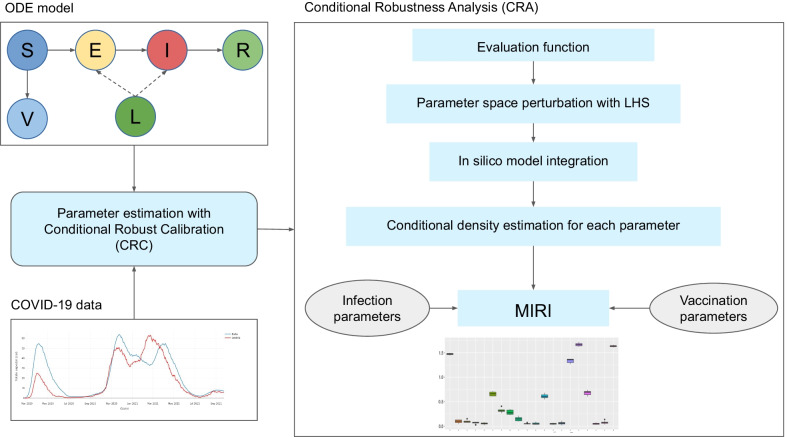
Graphic representation of the SEIRL-V model. Clinical stages for the population are: Susceptible (*S*), Exposed ($$E_{\nu }$$) where $$\nu =0,1,2$$ is the number of vaccine doses administered, Presymptomatic ($$P_{S,\nu }$$), Asymptomatic (*A*), Recovered (*R*), Mild infection (*M*), Severe infection (*H*), Critical infection (*ICU*), Dead (*D*), Vaccinated 1st dose ($$V_1)$$ and Vaccinated 2nd dose ($$V_2)$$ The intervention measures are represented by *L*

The SEIRL-V compartmental model used in this work was proposed in [12] and it is shown in Fig. 2. Susceptible individuals (class *S*) are vaccinated with two doses (classes *V*1 and *V*2). Both susceptibles and vaccinated, when in contact with infected people, can evolve into the exposed class (class $E_{\nu }$ with $\nu =0, 1, 2$ is the number of vaccine doses received), where they are infected but do not transmit the virus. Then, people in $E_{\nu }$ become presymptomatic (class $P_{S,\nu }$), where they can infect other people without showing symptoms. After the $P_{S,\nu }$ class, infected individuals are divided between asymptomatic (class *A*) and mild cases (class *M*). People in *M* can progress to hospitalization (class *H*) and then they may require treatment in ICU (class *ICU*). All infected classes can recover and evolve to the *R* class while only people in *H* and *ICU* class can die (class *D*). According to [18], immunity is acquired about 14 days after receiving the vaccine. Thus, we model classes $V_1$ and $V_2$ as those individuals who have received their first and second dose of vaccine 14 days before, respectively. Due to the rapid disease spread, we neglect births and non-epidemic related deaths. Thus, $S + E_{\nu } + P_{S,\nu } + A + M + H + ICU + R + D + V_1 + V_2=N$, where *N* is the total population. The systems of ODEs with an extensive description of the parameters can be found in Additional file 1 and in [11, 12]. Details on the calibration procedure CRC and its application to the SEIRL-V model can be found in Additional file 1.

### Conditional robustness analysis

The CRA is a method developed in Cancer Systems Biology to study complex signaling networks responsible of cancer cells proliferation and to discover their fragility. However, it is also suitable for a broader class of applications, once an ODE model is given in input. Indeed, it allows to identify those model parameters that have an high impact on a chosen output variable. By acting on these parameters, it is possible to yield the system toward the desired behavior. As shown in [11], the CRA was already employed to study the influence of COVID-19 related parameters on the hospitalization capacity. Here, the main purpose is to understand the impact of key parameters on the temporal evolution of hospitalization, in order to avoid another COVID-19 infection resurgence.

The main steps of the CRA are the following:

1. definition of an evaluation function $z_i$ representative of the property of interest, such as the area under the curve of the chosen observable $y_i$;
2. sampling of the parameter space using Latin Hypercube Sampling (LHS) and model integration to generate *in silico* evaluation functions. The parameter space is sampled starting from the parameter vector obtained in output from CRC;
3. definition of two thresholds $\alpha _i^L \ge 0$ and $\alpha _i^U \ge 0$ for selecting the simulated evaluation functions that are smaller and larger than the thresholds. Lets denote with $L(\alpha )=T(\alpha _i^L, z_{i,\mathbf{p} })$ and $U(\alpha )=T(\alpha _i^U, z_{i,\mathbf{p} })$ the two resulting sets of evaluation functions;
4. in the parameter space $P_S$, this selection induces the following subsets:
  1 $$\begin{aligned} P_{S,\alpha _i^L}= & {} \{ \mathbf{p} \in P_S: z_{i,\mathbf{p} } \in T(\alpha _i^L, z_{i,\mathbf{p }})) \}, \end{aligned}$$
  2 $$\begin{aligned} P_{S,\alpha _i^U}= & {} \{ \mathbf{p } \in P_S: z_{i,\mathbf{p }} \in T(\alpha _i^U, z_{i,\mathbf{p }}) \}. \end{aligned}$$
5. These subsets are then employed in the computation of the Moment Independent Robustness Indicator (MIRI) for each model parameter:
  3 $$\begin{aligned} {\mu _w}=\int |f_{p_w|L}(p_w)-f_{p_w|U}(p_w)| \mathrm {d}p_w \quad w=1,...,q, \end{aligned}$$
  where *q* is the total number of parameters.

We are interested in those parameters having an high MIRI value since it means that the corresponding conditional densities are well separated. As a result, choosing different parameter values of these parameters leads to opposite behaviors of the observed evaluation function. The CRA algorithm is run through the CRA Toolbox, a MATLAB package for performing robustness analysis given an ODE model in input [19]. Comprehensive details on the CRA and its applications are described in [11, 15, 20].

## Results

**Table 1:** Model parameters estimated by CRC for Italy using COVID-19 data from 1 September 2020 to 1 May 2021

Parameter	*p*_mode_	90th Percentile
$b_{e,1}$	0.1342	[0.1318–0.1721]
$b_{0,1}$	0.2109	[0.1833–0.214]
$b_{e,2}$	0.2512	[0.2393–0.3451]
$b_{0,2}$	0.19	[0.1366–0.205]
$b_1$	0.0120	[0.0112–0.0261]
$b_2$	0.0516	[0.0517–0.0677]
$b_3$	0.0145	[0.0112–0.0267
*FracSevere*	0.0253	[0.0221–0.0283]
$FracCritical_1$	0.005	[0.0051–0.0057]
$FracCritical_2$	0.007	[0.0061–0.0073]
$FracCritical_3$	0.0035	[0.0031–0.0039]
$FracCritical_4$	0.0048	[0.0041–0.0049]
*FracAsym*	0.4618	[0.4529–0.4772]
*IncubPeriod*	5.2668	[5.1188–5.2792]
*DurMildInf*	9.8982	[8.366–10.7008]
*DurAsym*	14.5149	[14.1736–15.7103]
*DurHosp*	15.6204	[15.0902–15.8961]
*TimeICUDeath*	12.6742	[12.0992–12.8822]
*ProbDeath*	34.7989	[33.4267–36.5895]
$ProbDeath_H$	25.3705	[22.1059–25.5674]
*PresymPeriod*	0.637	[0.6033–0.6363]
*n*	47.1717	[46.4324–58.8729
*K*	$4.44\cdot 10^4$	[$3.3381\cdot 10^4$ – $5.7296\cdot 10^4$]
$s_{01}$	1.0409	[1.0055–1.0442]
$s_{02}$	0.3095	[0.2653–0.369]
$s_{03}$	0.2634	[0.2099–03254]
$s_{04}$	0.6842	[0.612–0.7307]
$s_{05}$	0.1837	[0.1344–0.2704]

The second and third column show, respectively, the mode vector of $f_{\mathbf{P }|P_{S,\alpha ^L}}$ and the 90th percentile of the probability density function (pdf) of the parameter vector for Italy. Note that the pre-symptomatic period (PresymPeriod) is a percentage of the incubation period (IncubPeriod)

**Table 2:** Rate values of the model obtained by combining parameter values in Table 1 with the formulas shown in Additional file 1

Parameter	Value
$a_1$	0.0028
$a_0$	0.523
$g_1$	[0.0979, 0.0977, 0.0981, 0.0979]
$p_1$	[0.0031, 0.0033, 0.0029, 0.0031]
$p_2$	[0.0105, 0.0138, 0.0269, 0.0102]
$u_1$	0.0162
$g_2$	[0.0373, 0.034, 0.0209, 0.0376]
*u*	0.0274
$g_3$	0.0515
*f*	0.4618
$g_0$	0,0688

Rates $g_1$, $p_1$, $p_2$ and $g_2$ have multiple values which derive from the variation of the fraction of patients in ICU (*FracCritical*, see Additional file 1)

**Table 3:** Model parameters related to vaccination

Parameter	Value	*L*_1_	*U*_1_
$\delta$	240 days	180 days	540 days
$\rho _{1}$	0.8	0.3	0.95
$\rho _{2}$	0.95	0.6	0.95
$\tau$	21 days	21 days	104 days
$\rho _{3}$	0.808	0.3	0.95
$\rho _{4}$	0.946	0.6	0.95

The second column shows the nominal value of the parameter while the third and fourth columns report, respectively, the lower and upper boundaries of the sampling interval for parameter space perturbation in the CRA algorithm

**Fig. 3:**
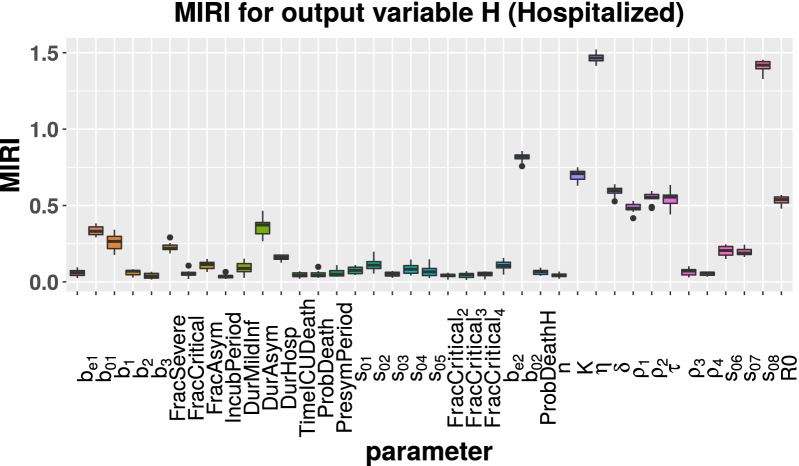
Boxplot of MIRI values for the 10 realizations of the CRA. The evaluation function is the area under the curve of H

**Fig. 4:**
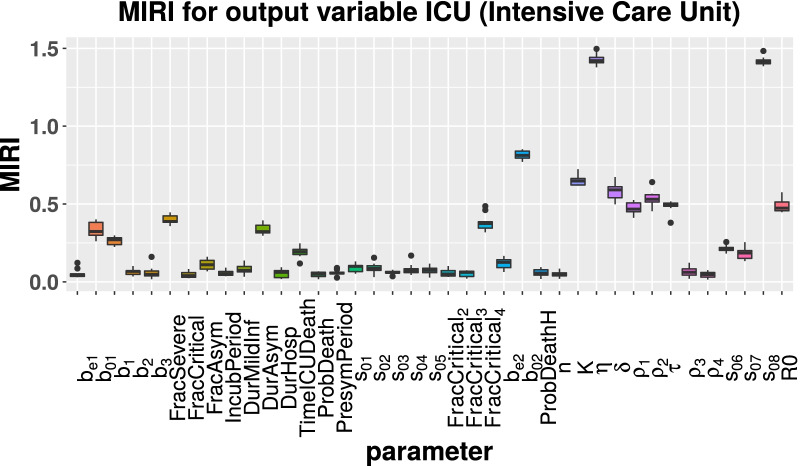
Boxplot of MIRI values for the 10 realizations of the CRA. The evaluation function is the area under the curve of ICU

**Fig. 5:**
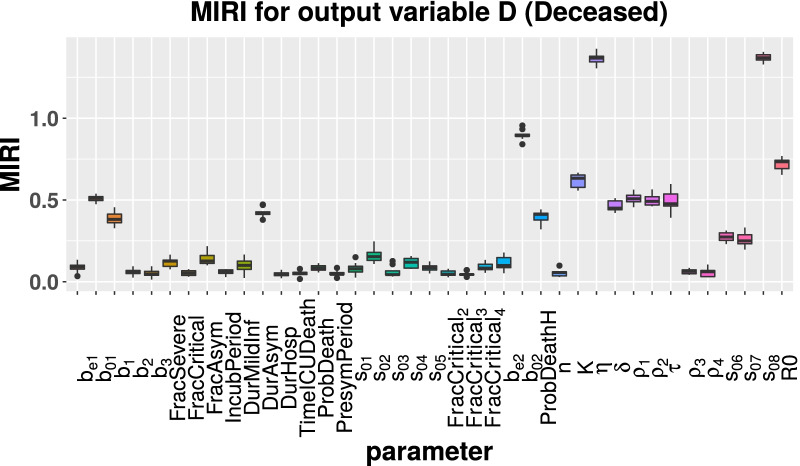
Boxplot of MIRI values for the 10 realizations of the CRA. The evaluation function is the area under the curve of D

Starting from the parameter vector estimated by CRC (see Tables 1 and 2), we run the CRA algorithm through the CRA Toolbox. We choose as evaluation function the area under the curve of H, ICU and D variables. The lower and upper boundaries of the sampling intervals for the parameter space are fixed equal to the 90-th percentile of the final probability density function (pdf) estimated by CRC (see Table 1). As regards vaccination parameters, the nominal values and the upper and lower boundaries are reported in Table 3. For these parameters, we choose wide ranges of variation in order to explore both optimistic and pessimistic scenarios. To run the CRA, we perturb the parameter space with Linear LHS generating $10^4$ samples and we set equal to 1000 the dimension of the upper and lower tail of the evaluation function pdf, in order to guarantee a stable estimation of the conditional parameter pdfs. We perform 10 realizations of the entire procedure to ensure invariance and stability of results. The temporal scenario analyzed spans 2 years from the 1st September 2021 until the 1st September 2023. With respect to model calibration, we introduce three intervention parameters ($s_{06}$, $s_{07}$ and $s_{08}$), in order to take into account the progressive relaxation of lockdown measures from Spring 2021. More in detail, on the 26 April 2021 most of the Italian regions returned to yellow zone, then on 24 May most activities, such as pools and gyms, were allowed to reopen. Finally, from 21 June the curfew was removed and nearly the entire country turned from yellow to white zone with almost no restrictions [21, 22]. During this period, the government maintained measures such as wearing protective masks, isolation of infected individuals through contact tracing and testing and household quarantining. The vaccination rate $\eta$ is perturbed between $10^5$ and $10^6$ first doses everyday, starting from 3 June 2021 when the vaccination campaign was opened to all people aged 16 and over [23]. Figures 3, 4, 5 show the MIRI returned by the CRA for all parameters while in the Additional file 1 the corresponding lower and upper parameter pdfs are reported. For all evaluation functions chosen, MIRI values are similar. In all cases, the two most influencing parameters are the vaccination rate $\eta$ and the intervention parameter $s_{08}$. Also other parameters related to vaccination, such as vaccine efficacy ($\rho _1$ and $\rho _2$), waning immunity ($\delta$) and time between the first and second dose ($\tau$), play a crucial role for controlling the pandemic. As expected, also the initial reproduction number $R_0$ has strong implications for controlling the hospitalization capacity. MIRIs highlights also the importance of asymptomatic transmission through parameters *DurAsym* and $b_{02}$, especially in the presence of highly infectious variants.

**Table 4:** Different parameter setups for model simulation

		$\eta$	$\delta$	$[s_{06},s_{07},s_{08}]$
Scenario A	1	$[10^5,10^6]$	[180d,540d]	[0.5, 0.6, 0.7]
	2	$[10^5,10^6]$	[180d,540d]	[0.8, 0.9, 1]
	3	$[10^5,10^6]$	[180d,540d]	[1.1, 1.2, 1.3]
Scenario B	1	$10^5$	[180d,540d]	[0.5, 1.5]
	2	$5 \cdot 10^5$	[180d,540d]	[0.5, 1.5]
	3	$10^6$	[180d,540d]	[0.5, 1.5]
Scenario C	1	$[10^510^6]$	180d	[0.5, 1.5]
	2	$[10^5,10^6]$	360d	[0.5, 1.5]
	3	$[10^5,10^6]$	540d	[0.5, 1.5]

In Scenario A, parameters $\eta$ and $\delta$ are perturbed in the intervals shown below while parameter vector [$s_{06}$,$s_{07}$,$s_{08}$] is fixed to three different values. In Scenario B and C parameters $\eta$ and $\delta$ are fixed, respectively. The unit of measurement for parameter $\delta$ is days (d)

**Fig. 6:**
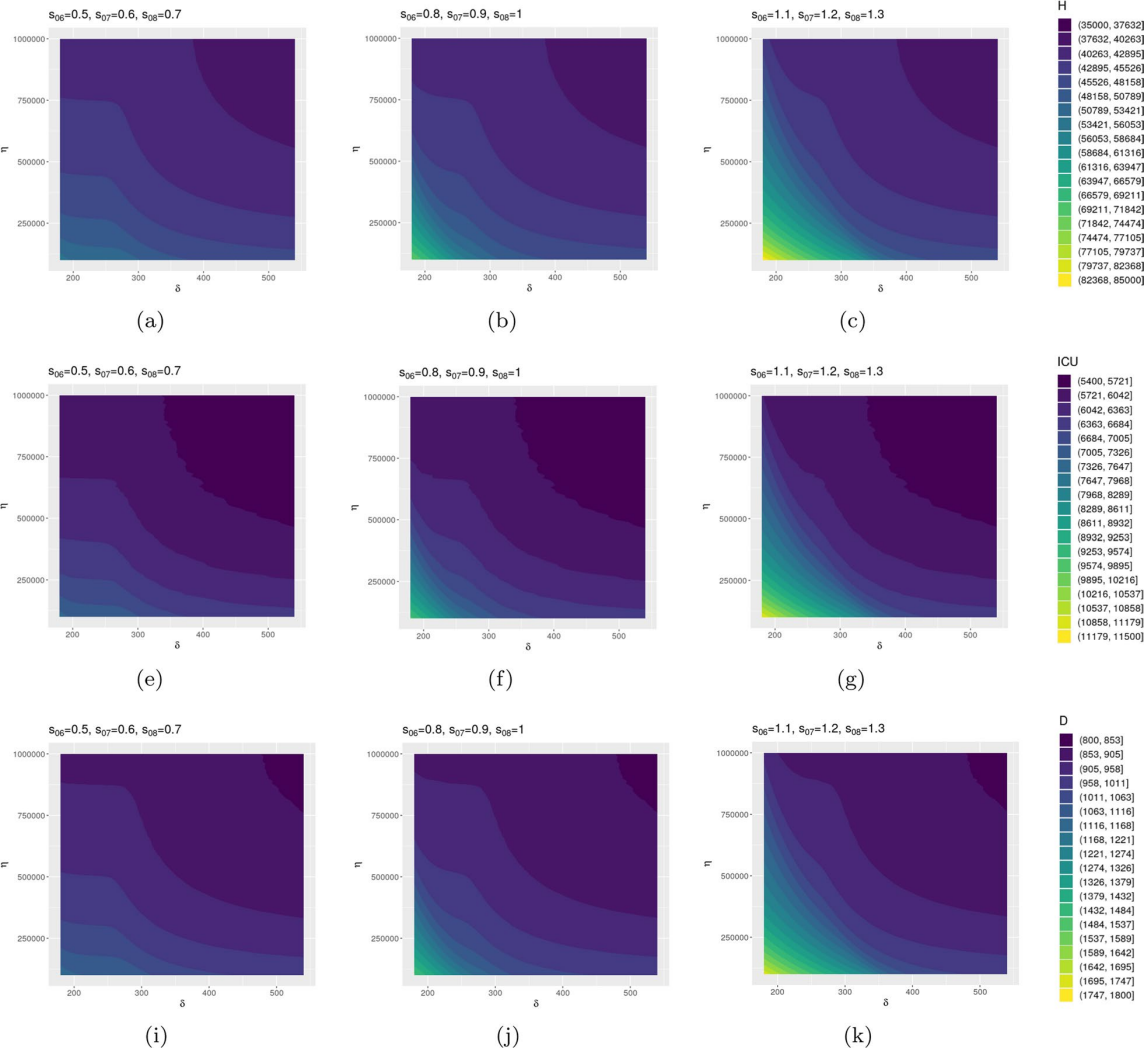
Italy. Results of Scenario A: perturbation of parameters $\eta$ and $\delta$. **(a–c).** Total number of hospitalization for three different values of [$s_{06}$,$s_{07}$,$s_{08}$].**(d–f).** Total number of ICU patients for three different values of [$s_{06}$,$s_{07}$,$s_{08}$].**(g–i).** Maximum number of deaths for three different values of [$s_{06}$,$s_{07}$,$s_{08}$]. Data are normalized over the Italian population ($\sim$ $60$ million) and multiplied by $10^5$

To explore possible epidemic dynamics based on the variation of these parameters, we perform further analysis for selected parameter setups. First of all, we focus on the vaccination rate, the waning immunity and the intervention parameters. As shown in Table 4, for each scenario we choose three different values for one parameter and we vary the remaining two in their entire interval. In each case, we simulate the model for a time span of 2 years and measure the total number of H and ICU patients and the maximum number of deaths. Figure 6 shows the results for Scenario A. The other two scenarios, B and C, are reported in Additional file 1. According to Figure 6, when high restrictions are enforced, the pandemic is under control even when the rate of vaccinations is low and immunity wanes in 6 months in the entire population. In case of medium and low levels of restrictions, if we increase the vaccination rate or the duration of immunity we can still have control over COVID-19 spread. These observations are confirmed by Additional file 1: Figs. S2 and S3 where we can see that, in case of rapidly decreasing immunity, a booster vaccination may be required.

**Fig. 7:**
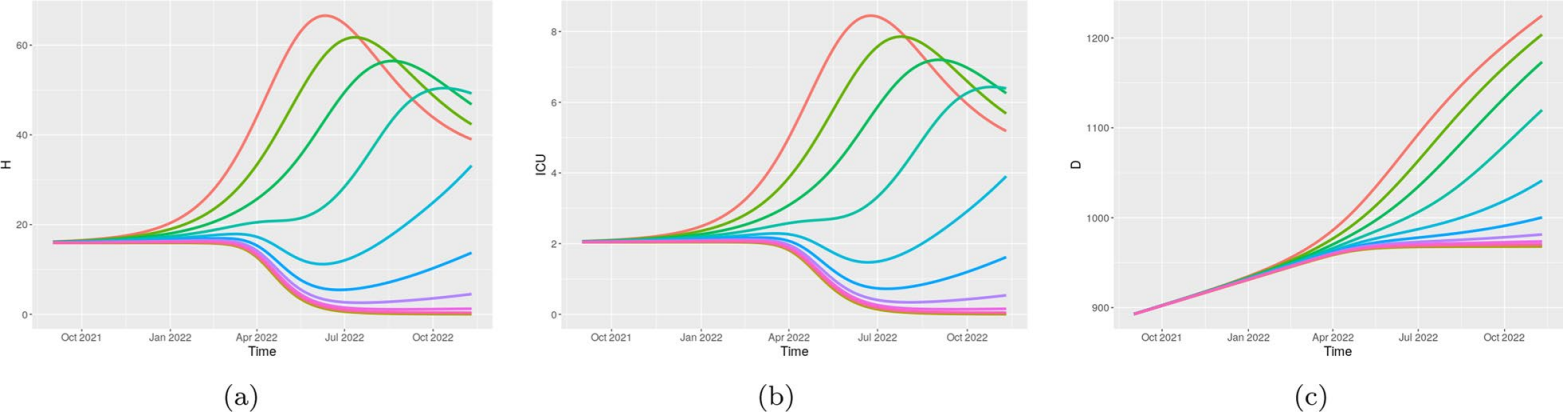
Italy. Predicted scenarios for different values of vaccine efficacy against infection. Parameter $\rho _1$, representing the efficacy of the first dose, is perturbed between 0.3 and 0.8 with a step size of 0.05. Parameter $\rho _2$, representing the efficacy of the second dose, is perturbed between 0.6 and 0.95 with a step size of 0.035. Higher values of $\rho _1$ and $\rho _2$ correspond to higher color curves of H, ICU and D

Finally, we investigate the role of average vaccine efficacy against transmission, since it is a highly debated parameter and a significant concern, especially in the presence of immune escape variants. Starting from CRC model calibration, we perturb parameters $\rho _{1}$ and $\rho _{2}$ in the intervals [0.3,0.8] and [0.6,0.95], respectively. We consider a moderate restriction scenario with $\eta =5 \cdot 10^5$ from June 2021 and [$s_{06}$,$s_{07}$,$s_{08}$]=[0.8, 0.9, 1]. Figure 7 shows how the time behavior of H, ICU and D changes with respect to these parameters. With an efficacy of the second dose of at least 75%, hospitalizations and deaths can be contained over the long period and an equilibrium with low case numbers is reached.

## Discussion

As SARS-CoV-2 is becoming endemic, a critical question for decision makers is understanding the best long-term strategies to contain its spread. Avoiding another wave is an essential mission, due to the deleterious consequences it causes not only in the healthcare sector but also from an economical and social point of view [24]. To provide insights into this key topic, we propose a computational framework derived from Cancer Systems Biology that, in combination with a SEIR model, can provide quantitative assessments on future scenarios about the evolution of the pandemic. The main characteristic of our approach is the use of conditional robustness and of the MIRI index to study key parameters influencing infection dynamics.

By combining model calibration and robustness analysis, we have found that parameters related to the vaccination are the most influencing ones for keeping under control the COVID-19 threat. MIRIs computed for H, ICU and D patients show that the progressive vaccination in reverse age order is one of the most effective strategies for decreasing subsequent deaths. Indeed, parameters $\eta$ and *K* have both MIRIs above 0.5. Moreover, parameters related to vaccine efficacy against infection ($\rho _{1}$ and $\rho _{2}$) have all MIRIs around 0.5, meaning that vaccines with higher efficacy, especially against new variants, provide better protection within the population. Duration of vaccination and natural immunity (parameter $\delta$) together with the gap between vaccine doses (parameter $\tau$) play also a crucial role in suppressing future outbreaks.

All approved COVID-19 vaccines are safe and highly effective against serious disease but, to date, vaccine longevity is an highly debated topic. Recent studies in Israel and Qatar show that effectiveness of the BNT162b2 vaccine (Pfizer-BioNTech) persists for 6 months after the second dose and then starts to decrease [25, 26]. In [27], the authors find that vaccine effectiveness against new infection declined from 91.8% to 75.0% in the population of New York between May and July 2021. The role of factors such as variations by age, health condition and variant circulation, is still uncertain. In Israel, it was shown that antibody levels decreased with age, especially in people 65 years of age or older and in people with immunosuppression. In Italy, the VP validity has been extended to 12 months since the second dose but the government has approved the administration of the third jab to people clinically vulnerable and over 80s, at least 6 months after the second dose [28]. Our results highlights that with waning immunity and low restrictions levels, re-vaccination is recommended to reduce the risk of hospitalization and deaths. Moreover, medium intervention measures can avoid resurgence of cases even with high waning immunity rate. To avoid new lockdowns that are no longer sustainable for financial stability and psychological burdens, a policy of test, trace and isolate together with temporary VPs can maintain control over the virus spread and keep case numbers low, in accordance with [29]. This is pivotal in order to disrupt asymptomatic chains of infections and detect vaccinated people that may have lost immunity. In a nutshell, even in the presence of the Delta variant and waning immunity, revaccination and contact tracing can compensate for the decreased effectiveness [10].

The workflow applied here is a combination of model calibration and robustness analysis of a SEIR model. First of all, parameter estimation allows to reproduce the current epidemic scenario from available data. Then, robustness analysis quantifies the impact of model parameter variations on severe cases requiring hospitalization. Once MIRIs are computed, this approach can be employed to study a variety of immune scenarios and possible interventions to alter the pandemic trajectory. Understanding pessimistic and optimistic outcomes is pivotal for government agencies which analyze how the epidemic may progress and how to reach a long-term disease equilibrium.

In this work we deliberately made some simplifying assumptions. Our model is a mean-field type of model where compartmentalization in age groups is not explicitly included, as it is common in this field [30]. However to take into account the reverse age order of the vaccination campaign, we introduce a dependency between hospitalized and immunized people (see Additional file 1). We also assume homogeneous spreading within the population, where all individuals are supposed to interact uniformly without distinctions by age and geographical region. Finally, we do not account for seasonality, which can affect the transmission of COVID-19 [31]. However, keeping these aspects simple is quite common when focusing on general spreading dynamics [10, 29, 32].

## Conclusions

In this study, we used a SEIR model to describe the time-varying nature of COVID-19 pandemic in Italy under multiple key factors, such as intervention measures and vaccinations. Our CRA algorithm suggests that controlling SARS-CoV-2 spreading without a new lockdown is possible in the medium and long term, through a policy based on booster vaccinations and testing. Large-scale vaccination is a key factor for hospitalization and mortality reduction and will be necessary until an endemic state for SARS-CoV-2 is reached.

Given a model representative of the factors of interest of the epidemic, our framework reveals particularly useful for a quantitative analysis of each pandemic phase. Moreover, the combination of CRC and CRA is fully generalizable and can be employed for identifying key parameters in conditioning a system to the wished behavior.

## Supplementary Information

**Additional file 1: **Supplementary Materials.

## Data Availability

The COVID-19 datasets generated and/or analysed during the current study are available in the COVID-19 Github repository of the Civil Protection Department of the Italian Government, https://github.com/pcm-dpc/COVID-19. The datasets about COVID-19 vaccines administered in Italy are available in the Github repository of the Italian Government, https://github.com/italia/covid19-opendata-vaccini. The code for running CRC and the ODE model of COVID-19 is available at https://github.com/fortunatobianconi/CRC. The code of the CRA Toolbox is available at http://gitlab.ict4life.com/SysBiOThe/CRA-Matlab.

## Abbreviations

SARS-CoV-2: Severe acute respiratory syndrome coronavirus 2
NPI: Non-pharmaceutical intervention
VOC: Variant of concern
VP: Vaccination pass
SIR: Susceptible-infected-recovered
ODE: Ordinary differential equation
CRA: Conditional robustness analysis
SEIR: Susceptible-exposed-infected-recovered
CRC: Conditional robust calibration
ICU: Intensive care unit
ABC-SMC: Approximate Bayesian computation sequential Monte Carlo
LHS: Latin hypercube sampling
MIRI: Moment independent robustness indicator
pdf: Probability density function.
