# Supplementary material for: Robustness analysis for quantitative assessment of vaccination effects and SARS-CoV-2 lineages in Italy
Source: BMC Infect Dis. 2022 Apr 29;22:415. doi: 10.1186/s12879-022-07395-2 (PMC9051820; doi:10.1186/s12879-022-07395-2)
Supplement: Supplementary file 1 — Additional file 1: Supplementary Materials. [file 12879_2022_7395_MOESM1_ESM.pdf]

Robustness analysis for quantitative assessment of vaccination effects and SARS-CoV-2 lineages in Italy

C. Antonini [1], S. Calandrini [1,2], F. Bianconi [3]

[1] *ICT4Life Srl, Perugia, Italy*
[2] *Department of Engineering, University of Perugia, Perugia, Italy*
[3] *COVID-19 epidemiological unit, Regional Government of Umbria, Perugia, Italy*

S1 SEIRL-V model

The system of ODEs describing the dynamics of COVID-19 is the following:

$$
\begin{aligned}
\dot{S} &= -(b_e P_{S,\nu} + b_0 A + b_1 M + b_2 H + b_3 ICU + \eta)S + \frac{R}{\delta} + \frac{V_2}{\delta} \\
\dot{E_\nu} &= (b_e P_{S,\nu} + b_0 A + b_1 M + b_2 H + b_3 ICU)S_\nu - a_0 E \\
\dot{P_{S,\nu}} &= a_0 E_\nu - a_1 P_{S,\nu} \\
\dot{A} &= z_\nu f a_1 P_{S,\nu} - g_0 A \\
\dot{M} &= (1 - z_\nu)(1 - f)a_1 P_{S,\nu} - g_1 M - p_1 M \\
\dot{H} &= p_1 M - g_2 H - p_2 H - u_1 H + \frac{1}{1 + (\frac{V_2}{K})^n} u_{vax} \\
\dot{ICU} &= p_2 H - g_3 ICU - uICU \\
\dot{R} &= g_0 A + g_1 M + g_2 H + g_3 ICU - \frac{R}{\delta} \\
\dot{D} &= uICU + u_1 H \\
\dot{V_1} &= \eta S - \frac{V_1}{\tau} - (b_e P_{S,\nu} + b_0 A + b_1 M + b_2 H + b_3 ICU)(1 - \rho_1)V_1 \\
\dot{V_2} &= \frac{V_1}{\tau} - (b_e P_{S,\nu} + b_0 A + b_1 M + b_2 H + b_3 ICU)(1 - \rho_2)V_2 - \frac{V_2}{\delta}
\end{aligned} \tag{1}
$$

It includes twelve compartments: susceptibles (S), exposed with $\nu = 0, 1, 2$ numbers of vaccines doses received (E_ν), pre-symptomatic with $\nu = 0, 1, 2$ vaccines doses received ($P_{S,\nu}$), asymptomatic (A), mild infected cases (M),hospitalized infected cases (H), hospitalized in ICU (ICU), recovered (R), dead (D) and vaccinated with one or two doses ($V1$ and $V2$). Variables V_1 and V_2 represent people vaccinated at least 14 days before. Variable S_ν with $\nu = 0, 1, 2$ is equal to: $S_0 = S$, $S_1 = (1 - \rho_1)V_1$ and $S_2 = (1 - \rho_2)V_2$. Parameters $b_i, \quad \forall i = e, 0, 1, 2, 3$, are the transmission rates of classes P_S, A, M, H, and ICU, respectively. Parameters $g_i \quad \forall i = 0, 1, 2, 3$ denote the different recovery rates of classes A, M, H, and ICU, respectively, while u_1 and u are the death rates of hospitalized and ICU people. Parameters $a_i \quad \forall i = 0, 1$ represent the rate of exit from classes E and P_S, while parameters p_1 and p_2 are the rate of admission to hospital and ICU, respectively. Parameter f is the fraction of asymptomatic individuals. Parameters related to vaccination are:

- η is the daily rate of the first dose of vaccine and it is modeled as a piecewise constant function;
- δ is the duration of natural and vaccine immunity;
- τ is the time between the first and second dose of vaccine;
- parameter $\rho_i \quad \forall i = 1, 2$ is the efficacy of the vaccine against infection;

- parameter $\rho_j \quad \forall j = 3, 4$ is the efficacy of the vaccine against hospitalization;
- parameter z_ν with $\nu = 0, 1, 2$ is introduced to represent vaccine efficacy against hospitalization. Thus, $z_0 = 0$, $z_1 = \rho_3$ and $z_2 = \rho_4$.

To model the prioritization of oldest age groups in the vaccination strategy, we introduce a Hill function between hospitalized (H) and immunized people (V_2), where K is the inverse feedback strength indicator and n is the Hill coefficient. The term u_{vax} is introduced to take into account the start of the massive vaccination campaign:

$$u_{vax} = \begin{cases} 0, & t < t_{vax} \\ 1, & t \geq t_{vax}, \end{cases} \tag{2}$$

where t_{vax} is January 10, 2021, i.e. 14 days after the first day of vaccination in Italy, December 27, 2020 [1]. We also include the multiple non-pharmaceutical interventions (NPIs) taken by the Italian government in order to contain the pandemic, such as protective masks, a policy of population-wide testing and contact tracing and a color coded system of restrictive measures. They are represented by parameter s_0 which multiplies the presymptomatic and asymptomatic transmission rates as follows:

$$\begin{aligned} b_{e,lock} &= b_e \cdot s_0 \\ b_{0,lock} &= b_0 \cdot s_0 \end{aligned} \tag{3}$$

We exclude parameters b_1, b_2, and b_3 because we assume that measures to contain the infectious capacity of these individuals have already been implemented from the beginning of the second wave of the epidemic. The transmission rate parameters of presymptomatic and asymptomatic infected are also influenced by the presence of new and highly transmissible variants. To take that into account, b_e and b_0 change as follows:

$$b_{e,lock} = \begin{cases} b_{e,1} \cdot s_0, & t < t_{var} \\ b_{e,2} \cdot s_0, & t \geq t_{var} \end{cases} \tag{4}$$

$$b_{0,lock} = \begin{cases} b_{0,1} \cdot s_0, & t < t_{var} \\ b_{0,2} \cdot s_0, & t \geq t_{var} \end{cases} \tag{5}$$

where t_{var} is the time of introduction of the new variants.

Model parameters are derived from the clinical observations through the following formulas:

- $a_1 = PresymPeriod^{-1}$
- $a_0 = (IncubPeriod - PresymPeriod)^{-1}$
- $g_1 = DurMildInf^{-1} \cdot (1 - FracSevere - FracCritical)$

- $p_1 = DurMildInf^{-1} - g_1$
- $p_2 = DurHosp^{-1} \cdot \frac{FracCritical}{(FracSevere+FracCritical)}$
- $u_1 = DurHosp^{-1} \cdot (\frac{(ProbDeathH \cdot \frac{FracSevere}{100})}{FracSevere}$
- $g_2 = DurHosp^{-1} - p_2 - u_1$
- $u = TimeICUDeath^{-1} \cdot (\frac{(ProbDeath \cdot \frac{FracCritical}{100})}{FracCritical}$
- $g_3 = TimeICUDeath^{-1} - u$
- $f = FracAsym$
- $g_0 = DurAsym^{-1}$,

where $IncubPeriod$ is the incubation period and $PresymPeriod$ is its corresponding infectious phase. $DurAsym$ is the average duration of asymptomatic infection and $DurMildInf$ is the average duration of mild symptoms or the time from symptom onset to hospitalization. The duration of severe infection $DurHosp$ is the time from hospital admission to recovery or death or ICU admission. $TimeICUDeath$ is the time from ICU admission to recovery or death. All time duration are expressed in days (d). $FracAsym$ is the percentage of infected people having asymptomatic infection while $FracSevere$ and $FracCritical$ are, respectively, the percentage of individuals requiring hospitalization and ICU-level care.

Using the next generation matrix, the formula for computing the basic reproduction number R_0, i.e., the number of individuals infected by a single infected individual during his infectious period, is [2, 3, 4]:

$$R_0 = N[\frac{b_e}{a_1} + f\frac{b_0}{g_0} + (1-f)\frac{1}{p_1+g_1}(b_1 + \frac{p_1}{p_2+g_2+u_1}(b_2 + b_3\frac{p_2}{u+g_3}))]. \quad (6)$$

S2 Conditional Robust Calibration (CRC)

Model parameters are estimated using the Conditional Robust Calibration (CRC) algorithm, which is a variant of the class of Approximate Bayesian Computation Sequential Monte Carlo (ABC-SMC) methods. CRC considers the model parameter vector as a random variable $\mathbf{P}$ with prior distribution $f_{\mathbf{P}}(\mathbf{p})$. Through an iterative procedure, it aims at approximating the posterior distribution $f_{\mathbf{P}|\mathbf{y}^*}(\mathbf{p})$, where $\mathbf{y}^*$ is the experimental dataset. In each iteration, CRC performs the following steps:

- sampling of the parameter space from a proposal distribution using Latin Hypercube Sampling (LHS), generating a number of samples N_S chosen by the user. For each parameter sample, the model is simulated to compute the output variables $\mathbf{y}$;

- the fitting between simulated and experimental data is measured through the definition of the following distance function:

$$d_{i,\mathbf{p}}(y_i, y_i^*) = \sum_{j=1}^{k} |y_i(t_j) - y_{ij}^*| \quad i = 1, ..., m, \tag{7}$$

  where $y_{i,j}^*$ is the *i-th* variable at time j of the experimental dataset and $y_i(t_j)$ is the simulated one;

- for each computed distance function, we define a threshold $\epsilon_i \geq 0$ which denotes the maximum accepted level of agreement between simulated and real data. This threshold allows to select a subset of parameter vectors P_{S,ϵ_i} for which the associated distance function satisfies this constraint;

- the parameter sets obtained for each output variable are intersected to obtain:

$$P_{S,\epsilon} = \{\bigcap_{i=1}^{m} P_{S,\epsilon_i}\}. \tag{8}$$

  Through a kernel density approach, we estimate $f_{\mathbf{P}|P_{S,\epsilon}}(\mathbf{p})$, an approximation of the target posterior distribution.

- The procedure can be repeated multiple times until the thresholds are sufficiently small. At each subsequent iteration, the initial proposal distribution for parameter sampling is shrunk around the mode of $f_{\mathbf{P}|P_{S,\epsilon}}(\mathbf{p})$.

A detailed description of CRC can be found in [5, 6, 7].

S3 CRC results: parameter estimation of the SEIRL-V model

CRC is applied to the ODE model in order to calibrate it using Italian COVID-19 data from 1 September 2020 to 1 May 2021. Data are taken from the Github repository of the Italian Civil Protection Department, which is updated daily with new positive cases, deaths and hospital occupancy [8]. Parameter estimation was performed against the everyday number of hospitalized, ICU and dead patients (H, ICU and D in the model), which are considered the most reliable measures. Indeed, under-counting of mild cases and fluctuations of new positive cases were frequent, especially during periods of high virus transmission.

According to [8], initial conditions of the model are set to $S_0 = 10^5 - E_0$, $E_{0,0} = (30000/N) \cdot 10^5$, $E_{1,0} = 0$, $E_{2,0} = 0$, $P_{S,0,0} = 20000$, $P_{S,1,0} = 0$, $P_{S,2,0} = 0$, $A_0 = 15000$, $M_0 = 26271$, $H_0 = 1437$, $ICU_0 = 109$, $R_0 = 208201$, $D_0 = 35497$, $V_{1,0} = 0$, $V_{2,0} = 0$. Data are normalized over the Italian population $N = 60 \cdot 10^6$ and multiplied by 10^5. Since September 2020, when a second wave of COVID-19 was just starting, the Italian government started to implement multiple containment measures. From November 2020 until April 2021,

Italian regions were divided in three risk zones on the basis of contagion data, with increasing level of restrictions. There are low-risk "yellow" zones with few restrictions, medium-risk "orange" zones where movements are allowed only in the same municipality and high-risk "red" zones where only essential movements are allowed.

In the model, we consider the most significant containment measures implemented by the Italian government and the most relevant events that may have accelerated the contagion, from the beginning of September 2020:

(1) 14 September 2020 ($T_{lock,1}$), school reopening;

(2) 6 November 2020 ($T_{lock,2}$), introduction of a three-tier color coded system of restrictive measures, based on the risk profile of each region;

(3) 24 December 2020 ($T_{lock,3}$), country-wide lockdown for Christmas holidays;

(4) 7 January 2021 ($T_{lock,4}$), school reopening and easing of some restrictions after the country-wide red area;

(5) 15 March 2021 ($T_{lock,5}$), removal of "yellow" zone in the color-coded system, leaving only medium and high risk zones.

Thus, the parameter vector representative of NPIs is $\boldsymbol{s}_0 = [s_{01}, s_{02}, s_{03}, s_{04}, s_{05}]$. It changes the transmission rate parameters as follows:

$$b_{e,lock} = \begin{cases} b_e, & t < T_{lock,1} \\ b_e \cdot s_{0,i}, & T_{lock,i} \leq t < T_{lock,i+1} \quad i = 1, ..., 4 \\ b_e \cdot s_{0,5} & t \geq T_{lock,5} \end{cases} \tag{9}$$

$$b_{0,lock} = \begin{cases} b_0, & t < T_{lock,1} \\ b_0 \cdot s_{0,i}, & T_{lock,i} \leq t < T_{lock,i+1} \quad i = 1, ..., 4 \\ b_0 \cdot s_{0,5} & t \geq T_{lock,5} \end{cases} \tag{10}$$

The time of introduction of new variants (t_{var}) is set equal to 19 January 2021, about a month before the rise of new infections. Moreover, to accurately simulate the epidemic in Italy, the fraction of patients in ICU is varied as follows: $FracCritical = FracCritical_1$ from day 0 (1 September 2020) to day 35 (5 October 2020), $FracCritical = FracCritical_2$ from day 36 to day 77 (16 November 2020), $FracCritical = FracCritical_3$ from day 78 to day 117 (26 December 2020), $FracCritical = FracCritical_4$ from day 118 onward. Vaccination parameters are set according to the Pfizer/BioNTech vaccine which is the most administered one in Italy [1]:

- parameter δ is set equal to 8 months (240 days), according to [9, 10]. We consider that natural immunity and immunity acquired through vaccination have on average the same duration [11];
- parameter τ is set to 21 days [12];

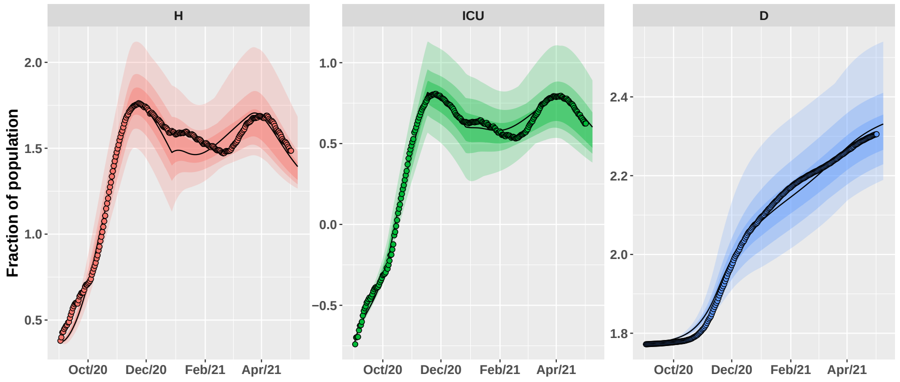

Figure S1: Italy.Time behavior of H, ICU, and D variables using as parameter vector the final mode vector computed by CRC (black line); dots are the public data available in [8]. Both data and simulations are in log-scale, normalized over the population of Italy (∼60 million) and multiplied by 10^5. The colored area represents the variation of the temporal behavior when the parameter vector varies between the 60th, 70th and 90th percentile of its conditional probability density function (pdf).

- parameter ρ_1 is set to 0.8 and parameter ρ_2 to 0.95 [13, 12];
- parameter ρ_3 is set to 0.808 and parameter ρ_4 to 0.946 [14, 15];
- vaccination rate η is modeled as a piecewise constant function and it is set in order to resemble the gradually increasing trend of first doses injected over time. It is set as $\eta = [0, 5.9 \cdot 10^{-4}, 0.0018, 0.0031, 0.0044]$ at days $[0, 132, 182, 219, 235]$ [16].

The parameter vector to estimate contains twenty-three model parameters and five interventions parameters, i.e., $\mathbf{p} \in \mathbb{R}^{28}$. As regards CRC, the number of samples in the parameter space is set to $N_S = 10^5$ and the number of iterations performed is 11. We repeat each iteration for 10 times, in order to ensure reliability of results. In table S1, the prior distribution of parameters is shown. As regards intervention parameters, we suppose a range of variation between 0.1 and 0.9 if the corresponding event is supposed to mitigate virus transmissibility while we suppose a range between 0.4 and 1.5 if the associated event correspond to lifting some NPIs. All the simulations are performed using Matlab (R2019a) on a Intel Core i7-4700HQ CPU, 2.40GHz 8, 16-GB memory, Ubuntu 18.04 LTS (64 bit).

S4 Additional results

For each model parameter, the CRA returns in output the so called Moment Independent Robustness Indicator (MIRI) index, which evaluates the shift be-

Table S1: Prior distribution of model parameter at the beginning of the first CRC iteration. The initial range of transmission rates is taken from [2]. Note that the pre-symptomatic period (PresymPeriod) is a percentage of the incubation period (IncubPeriod).

**Parameter**	**Prior**
$b_{e,1}$	log-U(0.01,1)
$b_{0,1}$	log-U(0.01,1)
$b_{e,2}$	log-U(0.01,1)
$b_{0,2}$	log-U(0.01,1)
b_1	log-U(0.001,1)
b_2	log-U(0.001,1)
b_3	log-U(0.001,1)
$FracSevere$	log-U(0.01,0.08)
$FracCritical_1$	log-U(0.001,0.02)
$FracCritical_2$	log-U(0.001,0.02)
$FracCritical_3$	log-U(0.001,0.02)
$FracCritical_4$	log-U(0.001,0.02)
$FracAsym$	U(0.2,0.7)
$IncubPeriod$	U(4,6)
$DurMildInf$	U(5,30)
$DurAsym$	U(5,20)
$DurHosp$	U(4,30)
$TimeICUDeath$	U(4,30)
$ProbDeath$	U(10,90)
$ProbDeath_H$	U(10,90)
$PresymPeriod$	log-U(0.5,0.9)
n	U(1,100)
K	U(1,10^5)
s_{01}	log-U(0.4,1.5)
s_{02}	log-U(0.1,0.9)
s_{03}	log-U(0.1,0.9)
s_{04}	log-U(0.4,1.5)
s_{05}	log-U(0.4,1.5)

tween the conditional densities $f_{p_w|L}(p_w)$ and $f_{p_w|U}(p_w)$ where $w = 1, ..., q$ is the number of model parameters and L and U are the lower and upper sets of evaluation functions . Higher is the MIRI value and larger is the shift between the two conditional probability density functions (pdfs). This means that a perturbation of the parameter space along the direction of the parameter having an high MIRI leads to a substantial variation of the evaluation function. Figures S2, S3 and S4 show the conditional parameter pdfs returned by the CRA for H variable while Figures S5, S6 and S7 report the parameter pdfs for ICU. Finally, Figures S8,S9 and S10 depict the pdfs for D. In blue we represent the 10 realizations of $f_{p_w|L}$ and in red the 10 realizations of $f_{p_w|U}$. The figures support and explain the MIRI boxplot in the main text. For instance, in Figure S2, parameters have almost all overlapping pdfs because their MIRI values are all around 0. On the other hand, parameters ρ_1, ρ_2 and s_{08} have a wide separation between the two distributions, which corresponds to a MIRI above 1 (see Figure 3 of the main text.)

Finally, Figures S11 and S12 show two other simulated scenarios when varying parameter δ, η and $[s_{06}, s_{07}, s_{08}]$.

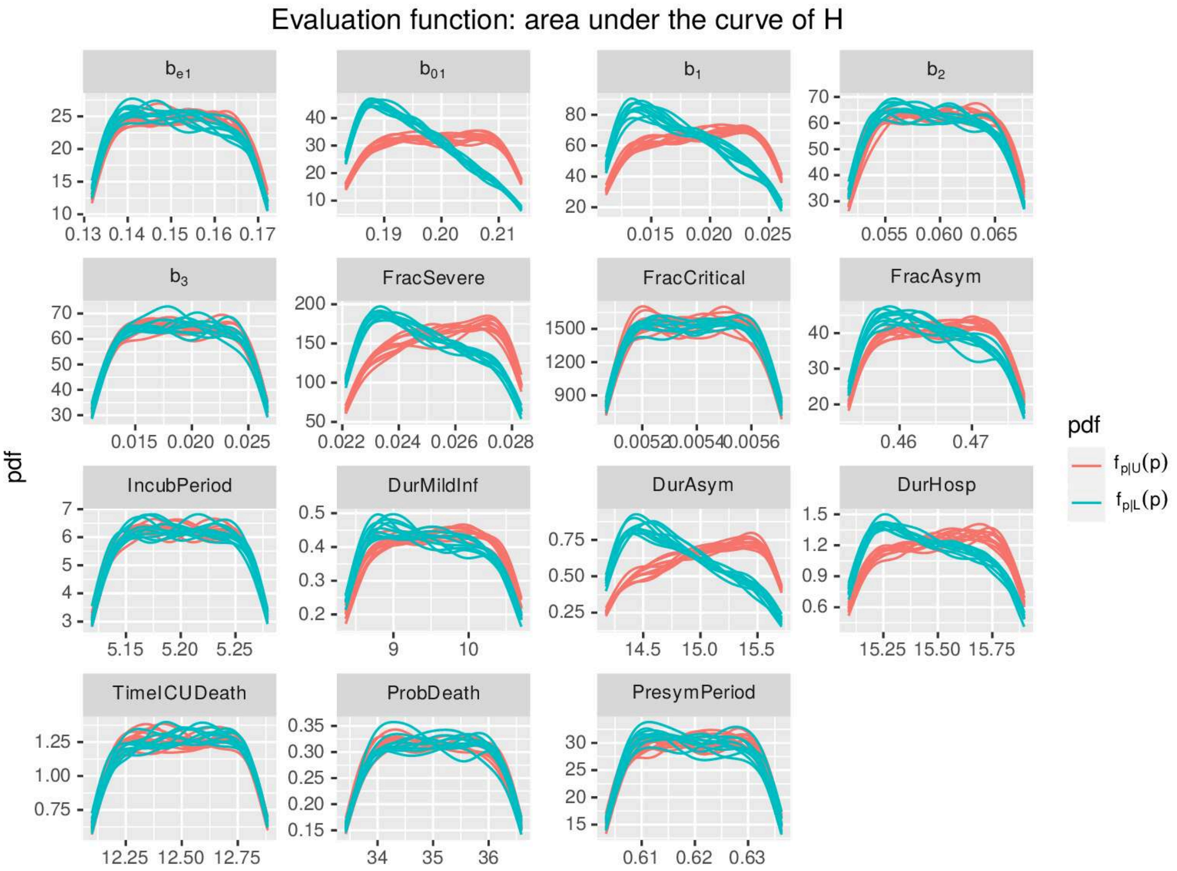

Figure S2: Conditional pdfs of model parameters after running the CRA algorithm using as evaluation function the area under the curve of H. Parameters with a great separation of the two pdfs are those with an higher MIRI value.

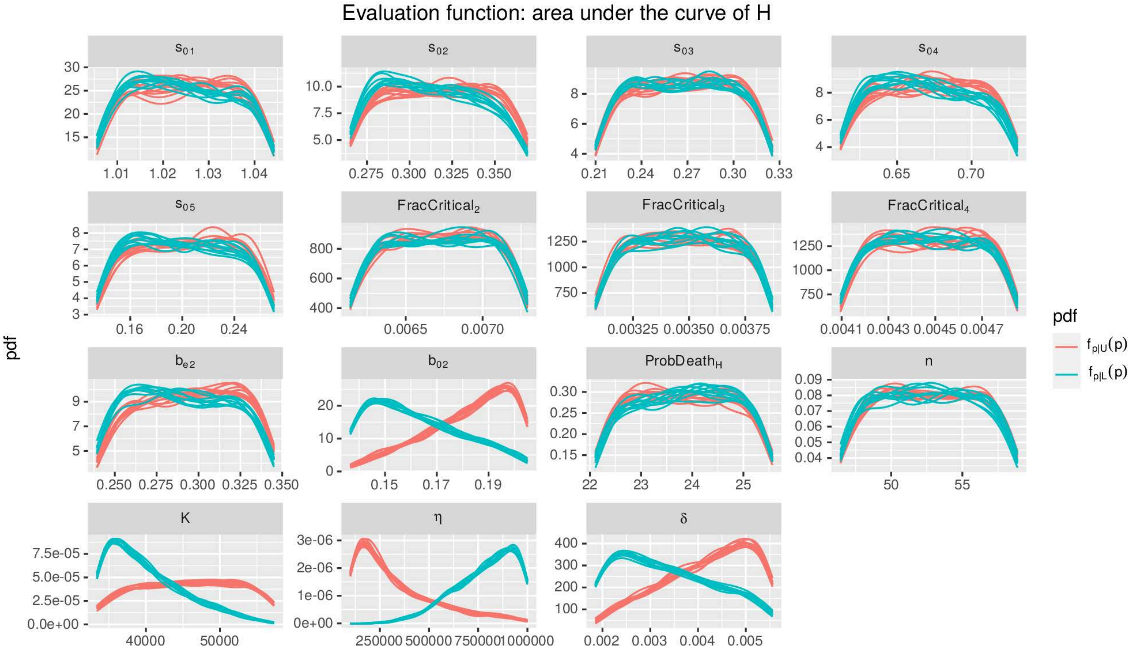

Figure S3: Conditional pdfs of model parameters after running the CRA algorithm using as evaluation function the area under the curve of H. Parameters with a great separation of the two pdfs are those with an higher MIRI value.

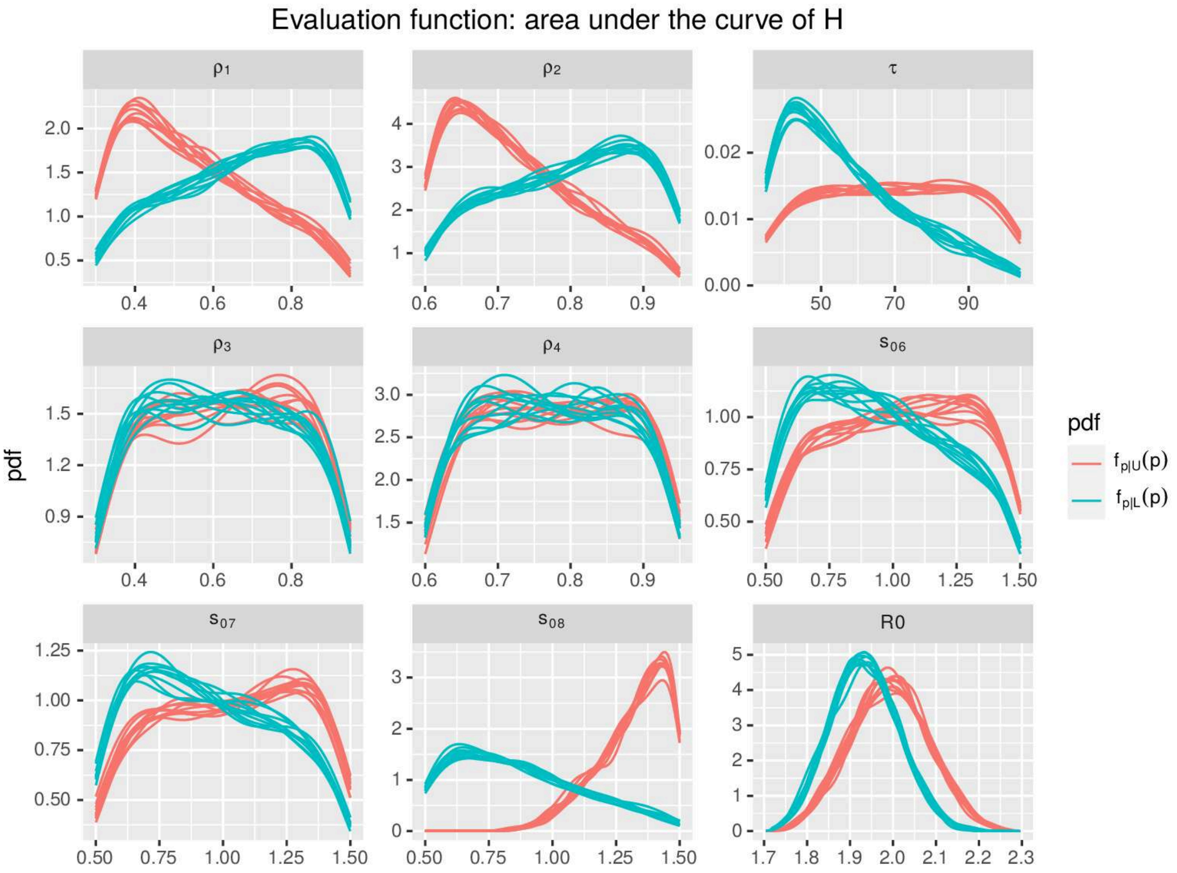

Figure S4: Conditional pdfs of model parameters after running the CRA algorithm using as evaluation function the area under the curve of H. Parameters with a great separation of the two pdfs are those with an higher MIRI value.

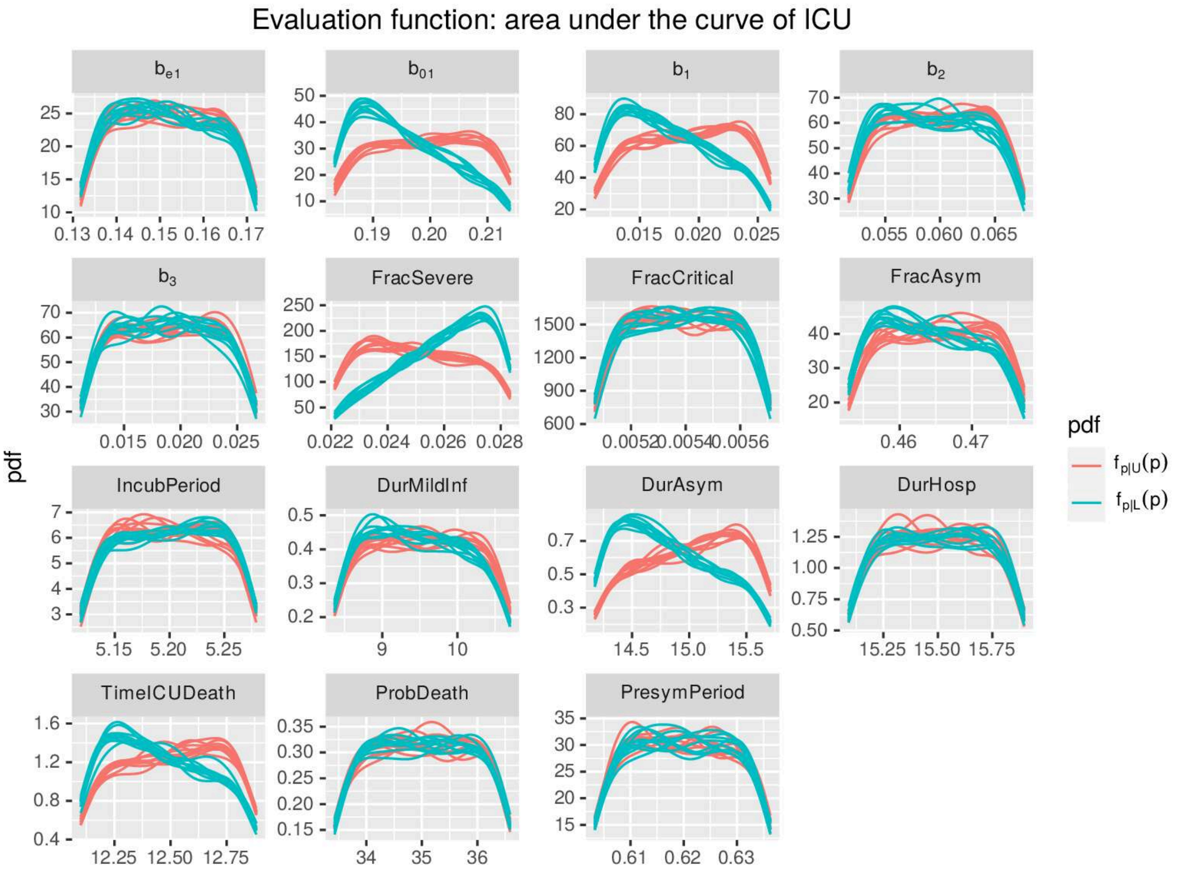

Figure S5: Conditional pdfs of model parameters after running the CRA algorithm using as evaluation function the area under the curve of ICU. Parameters with a great separation of the two pdfs are those with an higher MIRI value.

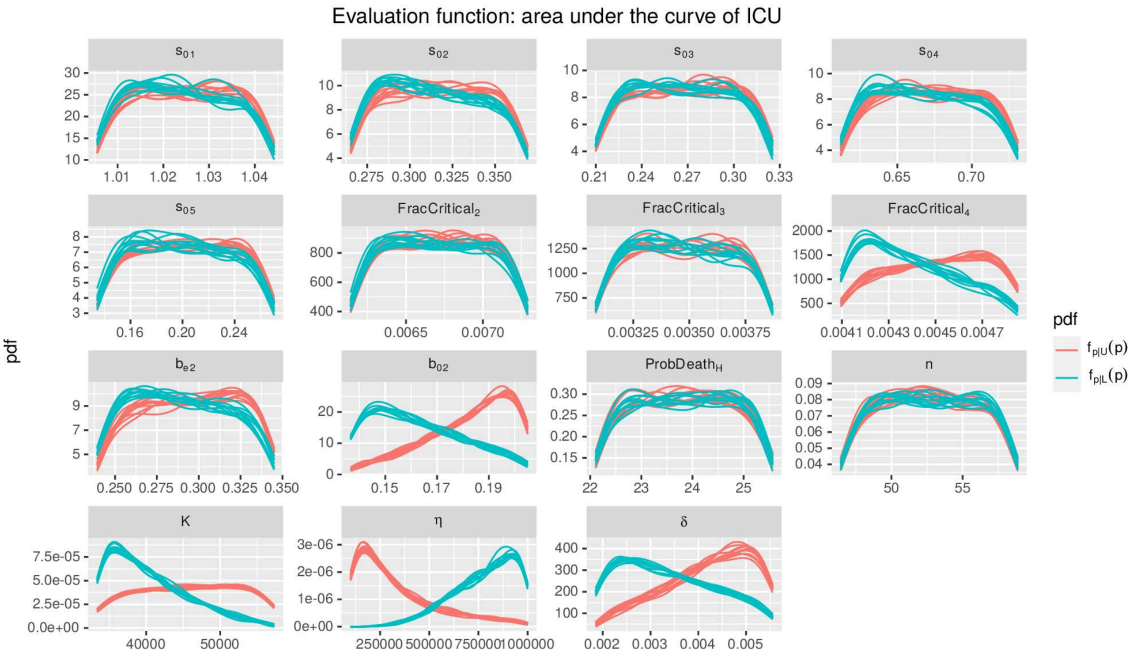

Figure S6: Conditional pdfs of model parameters after running the CRA algorithm using as evaluation function the area under the curve of ICU. Parameters with a great separation of the two pdfs are those with an higher MIRI value.

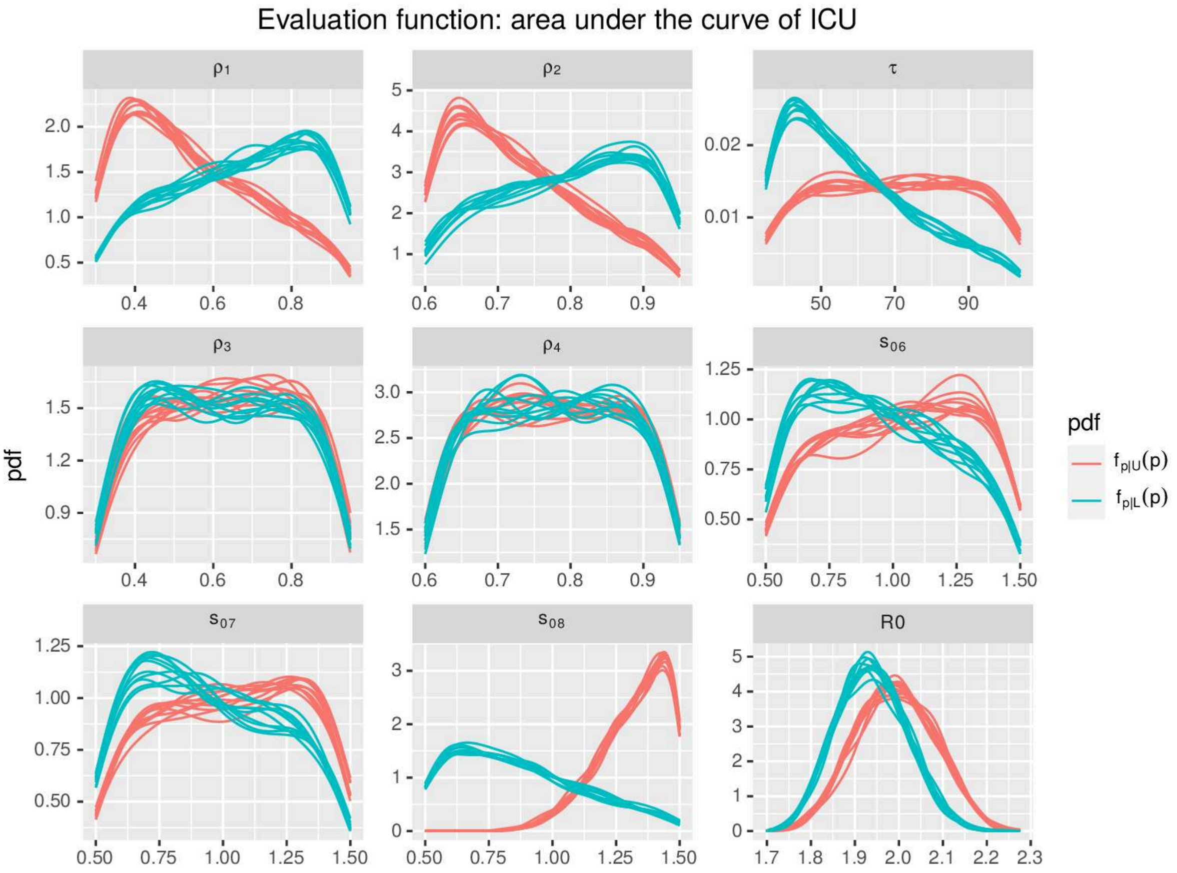

Figure S7: Conditional pdfs of model parameters after running the CRA algorithm using as evaluation function the area under the curve of ICU. Parameters with a great separation of the two pdfs are those with an higher MIRI value.

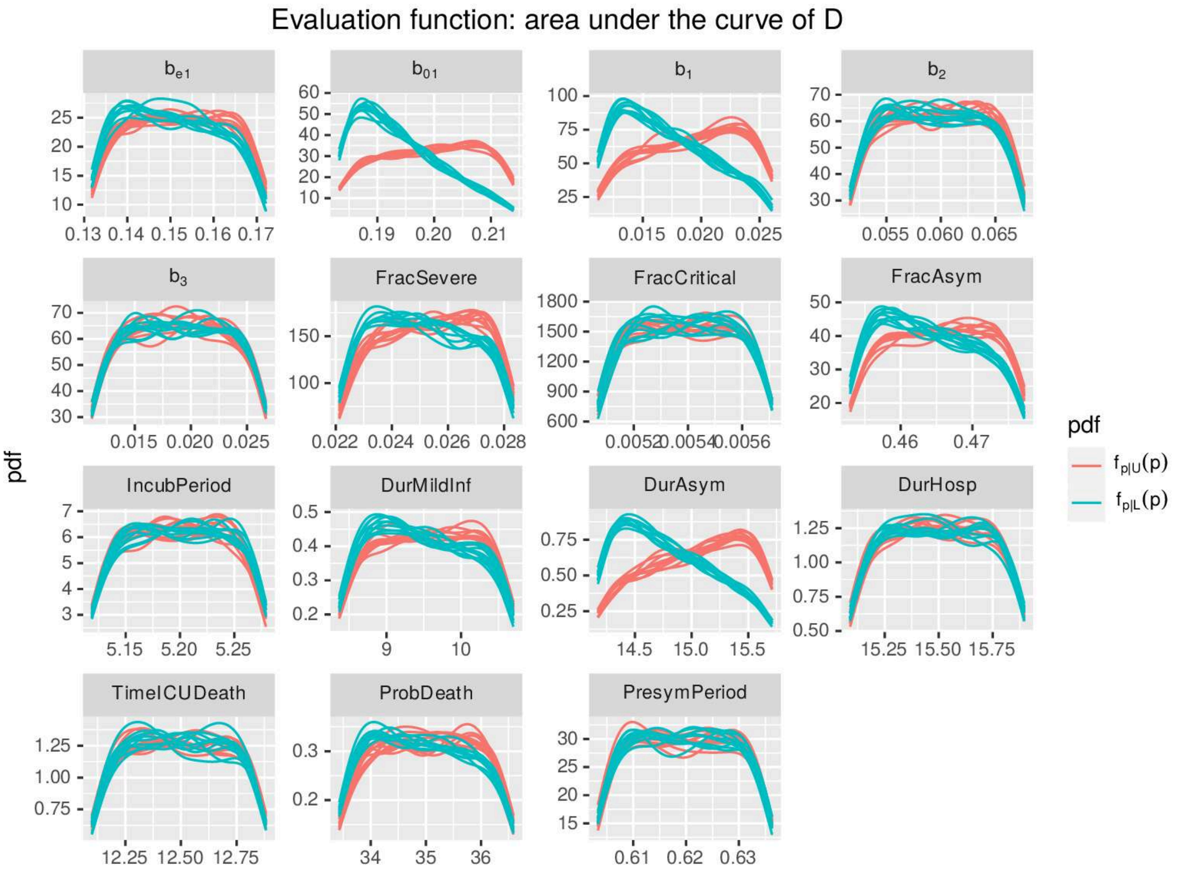

Figure S8: Conditional pdfs of model parameters after running the CRA algorithm using as evaluation function the area under the curve of D. Parameters with a great separation of the two pdfs are those with an higher MIRI value.

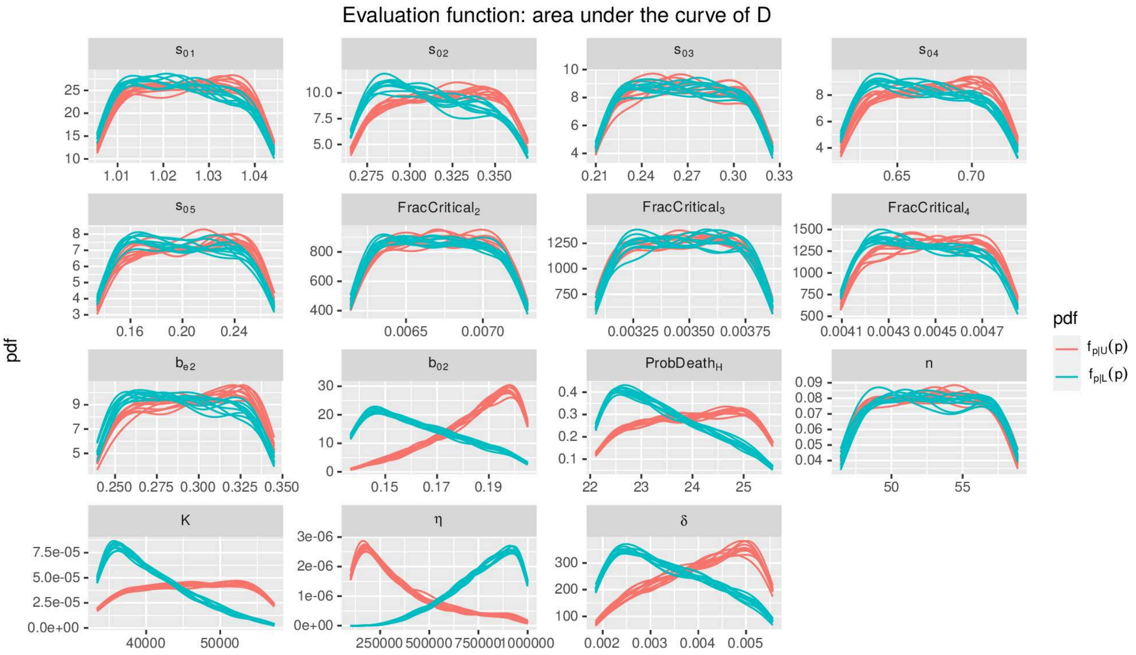

Figure S9: Conditional pdfs of model parameters after running the CRA algorithm using as evaluation function the area under the curve of D. Parameters with a great separation of the two pdfs are those with an higher MIRI value.

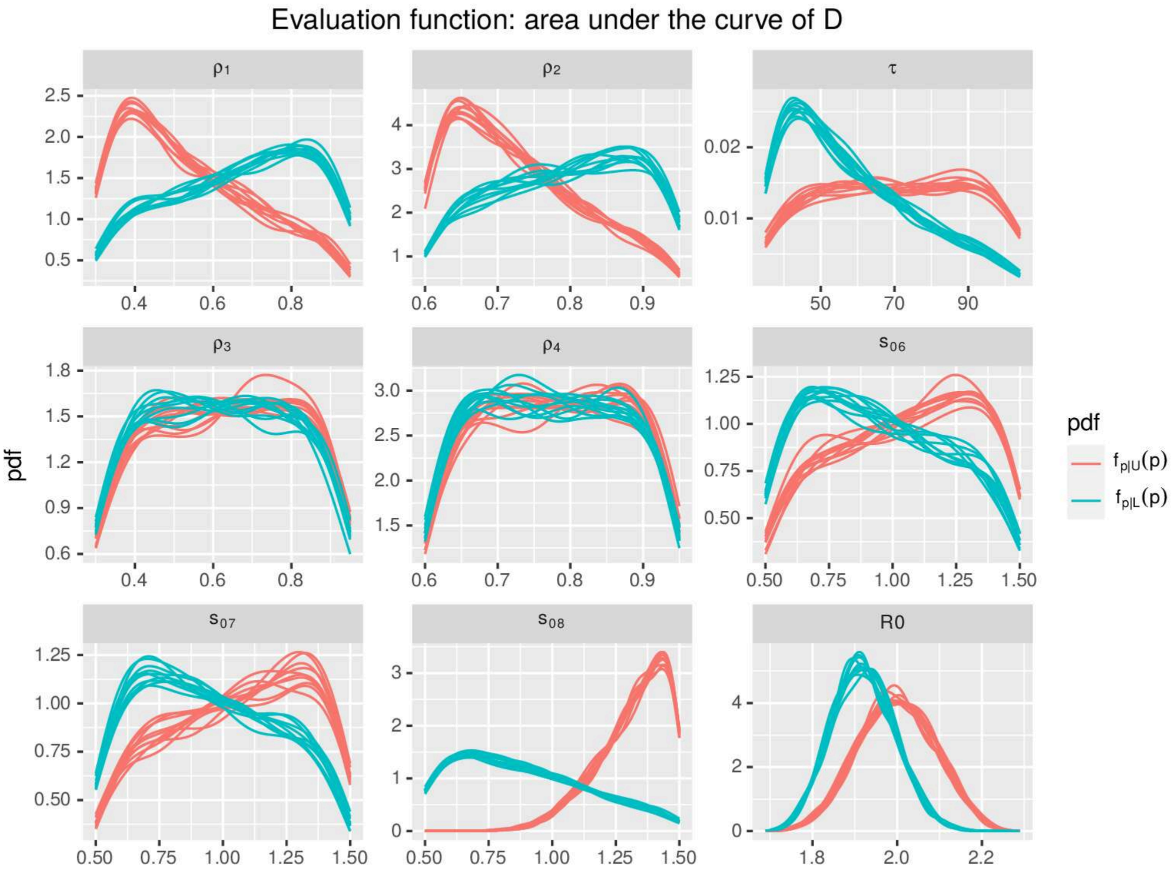

Figure S10: Conditional pdfs of model parameters after running the CRA algorithm using as evaluation function the area under the curve of D. Parameters with a great separation of the two pdfs are those with an higher MIRI value.

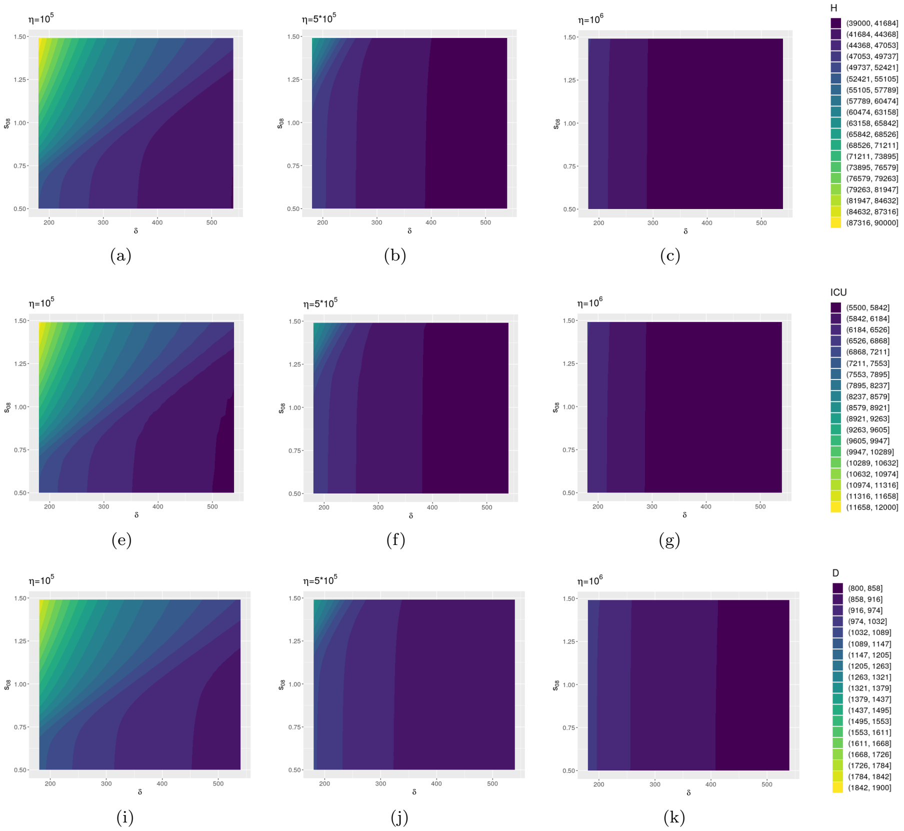

Figure S11: Results of Scenario B: perturbation of parameters δ and $[s_{06}$,s_{07},$s_{08}]$. **(a),(b) and (c).** Total number of hospitalization for three different values of η. **(d),(e) and (f).** Total number of ICU patients for three different values of η. **(g),(h) and (i).** Maximum number of deaths for three different values of η. Data are normalized over the Italian population ($\sim$60 million) and multiplied by 10^5.

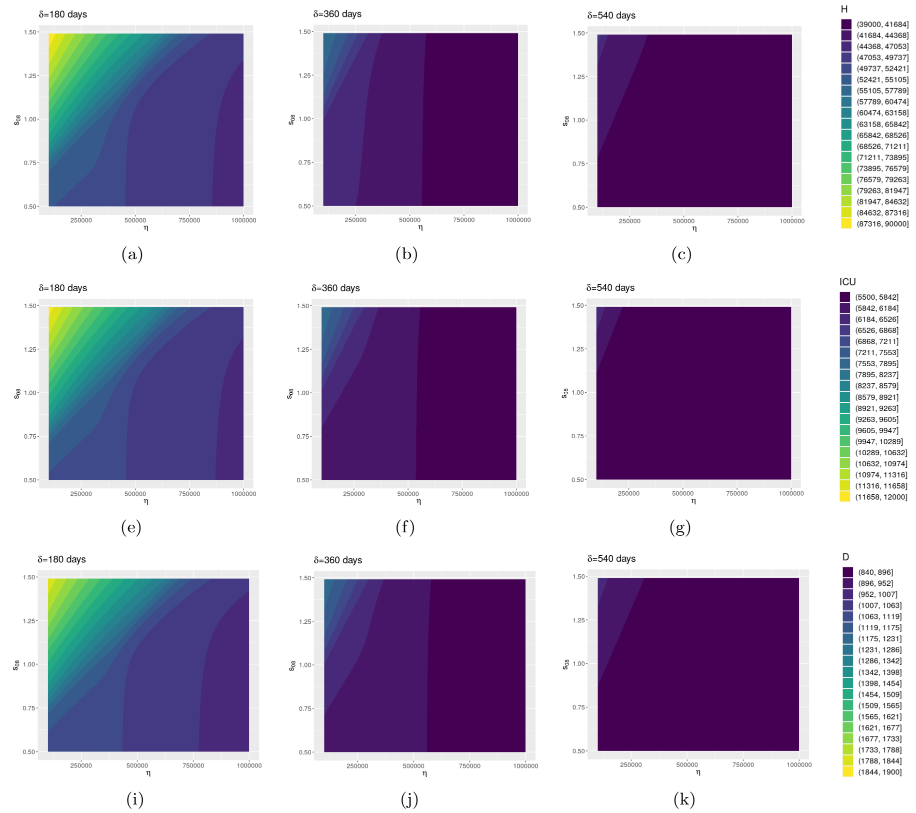

Figure S12: Results of Scenario C: perturbation of parameters η and $[s_{06}, s_{07}, s_{08}]$. **(a),(b) and (c).** Total number of hospitalization for three different values of δ. **(d),(e) and (f).** Total number of ICU patients for three different values of δ. **(g),(h) and (i).** Maximum number of deaths for three different values of δ. Data are normalized over the Italian population ($\sim$60 million) and multiplied by 10^5.

Bibliography

[1] Covid-19 opendata vaccini. https://github.com/italia/covid19-opendata-vaccini, 2021.

[2] Chiara Antonini, Sara Calandrini, Fabrizio Stracci, Claudio Dario, and Fortunato Bianconi. Mathematical modeling and robustness analysis to unravel covid-19 transmission dynamics: The italy case. *Biology*, 9(11):394, 2020.

[3] Giulia Giordano, Marta Colaneri, Alessandro Di Filippo, Franco Blanchini, Paolo Bolzern, Giuseppe De Nicolao, Paolo Sacchi, Patrizio Colaneri, and Raffaele Bruno. Modeling vaccination rollouts, sars-cov-2 variants and the requirement for non-pharmaceutical interventions in italy. *Nature Medicine*, pages 1–6, 2021.

[4] Gabriel Obed Fosu, Emmanuel Akweittey, and Albert Adu-Sackey. Next-generation matrices and basic reproductive numbers for all phases of the coronavirus disease. *Available at SSRN 3595958*, 2020.

[5] Fortunato Bianconi, Chiara Antonini, Lorenzo Tomassoni, and Paolo Valigi. Robust calibration of high dimension nonlinear dynamical models for omics data: An application in cancer systems biology. *IEEE Transactions on Control Systems Technology*, 2018.

[6] Fortunato Bianconi, Lorenzo Tomassoni, Chiara Antonini, and Paolo Valigi. A new bayesian methodology for nonlinear model calibration in computational systems biology. *Frontiers in Applied Mathematics and Statistics*, 6:25, 2020.

[7] Fortunato Bianconi, Chiara Antonini, Lorenzo Tomassoni, and Paolo Valigi. Application of conditional robust calibration to ordinary differential equations models in computational systems biology: a comparison of two sampling strategies. *IET Systems Biology*, 14(3):107–119, 2020.

[8] Dipartimento protezione civile, github repository. https://github.com/pcm-dpc/COVID-19, 2021.

[9] Stefania Dispinseri, Massimiliano Secchi, Maria Franca Pirillo, Monica Tolazzi, Martina Borghi, Cristina Brigatti, Maria Laura De Angelis, Marco

Baratella, Elena Bazzigaluppi, Giulietta Venturi, et al. Neutralizing antibody responses to sars-cov-2 in symptomatic covid-19 is persistent and critical for survival. *Nature Communications*, 12(1):1–12, 2021.

[10] Pfizer and biontech initiate a study as part of broad development plant o evaluate covid-19 booster and new variants. `https://www.pfizer.com/news/press-release/press-release-detail/pfizer-and-biontech-initiate-study-part-broad-development`, 2021.

[11] Tyll Krueger, Krzysztof Gogolewski, Marcin Bodych, Anna Gambin, Giulia Giordano, Sarah Cuschieri, Thomas Czypionka, Matjaz Perc, Elena Petelos, Magdalena Rosińska, et al. Risk of covid-19 epidemic resurgence with the introduction of vaccination passes. *medRxiv. The Preprint Server for Health Sciences*, 2021.

[12] Fernando P Polack, Stephen J Thomas, Nicholas Kitchin, Judith Absalon, Alejandra Gurtman, Stephen Lockhart, John L Perez, Gonzalo Pérez Marc, Edson D Moreira, Cristiano Zerbini, et al. Safety and efficacy of the bnt162b2 mrna covid-19 vaccine. *New England Journal of Medicine*, 383(27):2603–2615, 2020.

[13] Mark G Thompson, Jefferey L Burgess, Allison L Naleway, Harmony L Tyner, Sarang K Yoon, Jennifer Meece, Lauren EW Olsho, Alberto J Caban-Martinez, Ashley Fowlkes, Karen Lutrick, et al. Interim estimates of vaccine effectiveness of bnt162b2 and mrna-1273 covid-19 vaccines in preventing sars-cov-2 infection among health care personnel, first responders, and other essential and frontline workers—eight us locations, december 2020–march 2021. *Morbidity and Mortality Weekly Report*, 70(13):495, 2021.

[14] Task force covid-19 del dipartimento malattie infettive e servizio di informatica, istituto superiore di sanità. epidemia covid-19. aggiornamento nazionale: 14 luglio 2021. `https://www.epicentro.iss.it/coronavirus/bollettino/Bollettino-sorveglianza-integrata-COVID-19_14-luglio-2021.pdf`, 2021.

[15] Alberto Mateo-Urdiales, Stefania Spila Alegiani, Massimo Fabiani, Patrizio Pezzotti, Antonietta Filia, Marco Massari, Flavia Riccardo, Marco Tallon, Valeria Proietti, Martina Del Manso, et al. Risk of sars-cov-2 infection and subsequent hospital admission and death at different time intervals since first dose of covid-19 vaccine administration, italy, 27 december 2020 to mid-april 2021. *Eurosurveillance*, 26(25):2100507, 2021.

[16] Chiara Antonini, Sara Calandrini, and Fortunato Bianconi. A modeling study on vaccination and spread of sars-cov-2 variants in italy. *Vaccines*, 9(8):915, 2021.
